# Revision surgery after failure of lateral unicompartmental knee replacement with a mobile-bearing device: a retrospective non -designer case-series

**DOI:** 10.1007/s00402-025-05822-y

**Published:** 2025-03-21

**Authors:** Mustafa Hariri, Jakob Freytag, Kevin-Arno Koch, Paul Mick, Timo Nees, Kevin Knappe, Tobias Renkawitz, Tilman Walker

**Affiliations:** Department of Orthopaedics, Heidelberg University Hospital, Schlierbacher Landstrasse 200a, 69118 Heidelberg, Germany

**Keywords:** Lateral unicompartmental knee replacement, UKA, UKR, Mobile bearing, Revision arthroplasty

## Abstract

**Introduction:**

Failure of lateral unicompartmental knee replacement (UKR) with a mobile-bearing (MB) device often occurs due to bearing dislocation. The effectiveness of various treatment options for revision surgery is not clear. Therefore, the purpose of this study was to report on the failure modes in lateral MB-UKR, as well as the results of different revision strategies.

**Materials and methods:**

Patients who experienced failure of lateral MB-UKR and required revision surgery at a single-center between 2008 and 2020 were included in this retrospective study. The aim of the study was to report the reasons for failure and to document all treatment strategies employed. Survivorship analysis was conducted using the Kaplan-Meier estimator, with the endpoint defined as ‘re-revision for any reason’. Survival rates among various treatment strategies were compared using the log-rank test.

**Results:**

A total of 13 patients were included in the study, with a mean follow-up (FU) period of 94.7 ± 36.4 months. The reasons for failure included bearing dislocation in 69.2%, progression of osteoarthritis (OA) in 23.1%, and periprosthetic joint infection in 7.7%. Treatment options included replacement of the tibial component with a fixed-bearing (FB) device in 23.1% of cases, solitary exchange of the bearing in 53.8%, or conversion to an unconstrained total knee replacement (TKR) in 23.1%. The re-dislocation rate in patients who underwent a bearing exchange as a treatment for bearing dislocation was 100%, with a mean FU period of 8.4 ± 11 months. Therefore, survivorship for the treatment of bearing dislocation differed significantly in these patients compared to those who received an exchange to a FB device (0% vs. 100%, *p* = 0.014).

**Conclusions:**

Addressing the recurring issue of bearing dislocation in lateral MB-UKR demands a more comprehensive approach than merely replacing the bearing. Effective solutions include replacing the tibial component with an FB design or converting to an unconstrained TKR.

**Level of evidence:**

Retrospective cohort study, Level IV.

## Introduction

Lateral unicompartmental knee replacement (UKR) is a surgical procedure that has gained popularity in the treatment of isolated lateral compartment osteoarthritis (OA). This minimally invasive procedure offers the advantages of pain relief, improved knee function, and quicker recovery compared to total knee replacement [1]. Isolated OA of the lateral knee compartment is up to ten times less common than that of the medial compartment [2], making lateral UKR rare and accounting for only 2% of all UKR [3].

Recent studies have demonstrated the lowest revision rates for fixed-bearing (FB) designs in lateral UKR [3, 4]. While OA progression and aseptic loosening are common revision causes for both designs, bearing dislocation is a main concern of lateral UKR with a mobile-bearing (MB) design [5]. Even with the most advanced surgical techniques and prosthesis designs in lateral MB-UKR, bearing dislocation remains a major cause of early failure [6]. To date there is no consensus on the optimal treatment for these patients.

Reported revision strategies for bearing dislocation in lateral UKR beside conversion to TKR include replacement of the bearing or the whole tibial component to a FB design [7, 8]. The effectiveness of the aforementioned revision strategies has only been reported by the developer center [7].

Thus, the aim of the current study was to report failure modes in lateral MB-UKR and the results of different revision strategies after failed lateral MB-UKR in a non-designer center. We hypothesized that revising to a FB design is a safe procedure with better results than an isolated bearing exchange.

## Materials and methods

### Ethical approval

was obtained from the institutional review boards of the University of Heidelberg (S-944-2021) and the study was conducted in accordance with the Helsinki Declaration of 1975, as revised in 2013. Informed consent was obtained from all participating patients.

The current study retrospectively analyzes prospectively collected data from a series of patients who underwent revision surgery after failure of lateral MB-UKR between 2008 and 2020. In all index surgeries the Oxford Domed Lateral prosthesis (Zimmer Biomet Inc., Warsaw, Indiana, USA) was used.

The primary indication for index surgery was severe osteoarthritis of the lateral compartment with full thickness articular cartilage loss (“bone-on-bone”). In all cases, the anterior cruciate ligament (ACL) as well as the medial (MCL) and lateral collateral ligaments (LCL) were functionally intact, the valgus deformity was manually correctable to ensure that no ligaments were rigid and there was no evidence of OA in the medial compartment on varus stress radiographs. OA of the patellofemoral joint was not considered a contraindication unless there was a deep eburnation or bone grooving on the medial facet of the patella. Rheumatoid arthritis, fixed valgus deformity, previous osteotomy, or a flexion deformity > 15° were considered contraindications. All surgeries were performed using a minimally invasive surgical technique (MIS) through a lateral parapatellar approach without dislocation of the patella. Internal rotation of the tibial plateau and anatomical positioning of the femoral component were considered to avoid elevation of the joint line. Bearing thickness was selected in full extension. Depending on the bone quality, the use of a cemented or uncemented fixation of the femoral component was chosen, whereas the tibial component was always cemented in both groups [6].

All revision surgeries were conducted at the same university hospital. They were performed through the initial approach by three senior surgeons with high experiences in revision knee arthroplasty. In one case the index surgery as well as re-revision was performed in a different hospital and all information regarding the index surgery as well as re-revision were obtained from the responsible orthopedic surgeon. In all other patients the index surgery as well as further revision surgeries were performed at the same university hospital.

The surgical procedures used for revision was depending on the cause of revision and included the following:

1. Replacement of the tibial component with a FB design as described previously [8]. There were two different tibial components used: Oxford Fixed Lateral (Zimmer Biomet, Warsaw, Indiana, USA) and Vanguard M Partial (Zimmer Biomet, Warsaw, Indiana, USA). Both components were compatible with the remained femoral component.
2. Isolated replacement of the bearing with a bearing of similar size or thicker in case of a loose flexion gap.
3. Conversion to an unconstrained TKR using the P.F.C. Total Knee System (DePuy Synthes, Warsaw, Indiana, USA).

To present an overview of the health status in the current study group, the Charlson comorbidity index was used [9].

Radiographic analysis was performed with the use of standardized postoperative radiographs that were aligned with fluoroscopic control to obtain views parallel to the tibial component in the AP-view and parallel to the femoral component in the lateral view. The radiographs were analyzed by two independent examiners (MH, JF) focusing on progression of OA or signs of loosening.

### Statistical analysis

Data were collected and analyzed using SPSS version 29.0 (SPSS Inc., Chicago, IL). The primary endpoint was “re-revision” defined as any surgery in which at least one of the components was replaced. Survivorship analysis was performed with the Kaplan-Meier estimator. Survival rates were compared using the log rank test.

The empirical distribution of continuous variables was described using mean and standard deviation (SD).

For all tests, the significance level was set at *p* < 0.05.

## Results

A total of thirteen patients were included in the analysis. No patient was lost to follow-up and five patients died during the study period for unrelated reasons. All demographic data are shown in Table 1.

**Table 1:** Demographics of the study population

Number of patients	13
Gender	
Female	9 (69.2%)
Male	4 (30.8%)
Side	
Right	9 (69.2%)
Left	4 (30.8%)
Age at time of revision surgery	
Mean	67.2
SD	± 9.1
Time to index revision surgery (months)	
Mean	32.2
SD	± 26.8
BMI (kg/m^2^)	
Mean	27.1
SD	± 6.1
Follow-up (months)	
Mean	94.7
SD	± 36.4
Charlson comorbidity Index	
Mean	2.7
SD	± 1.3

SD: Standard deviation; BMI: Body-mass-index

**Fig. 1:**
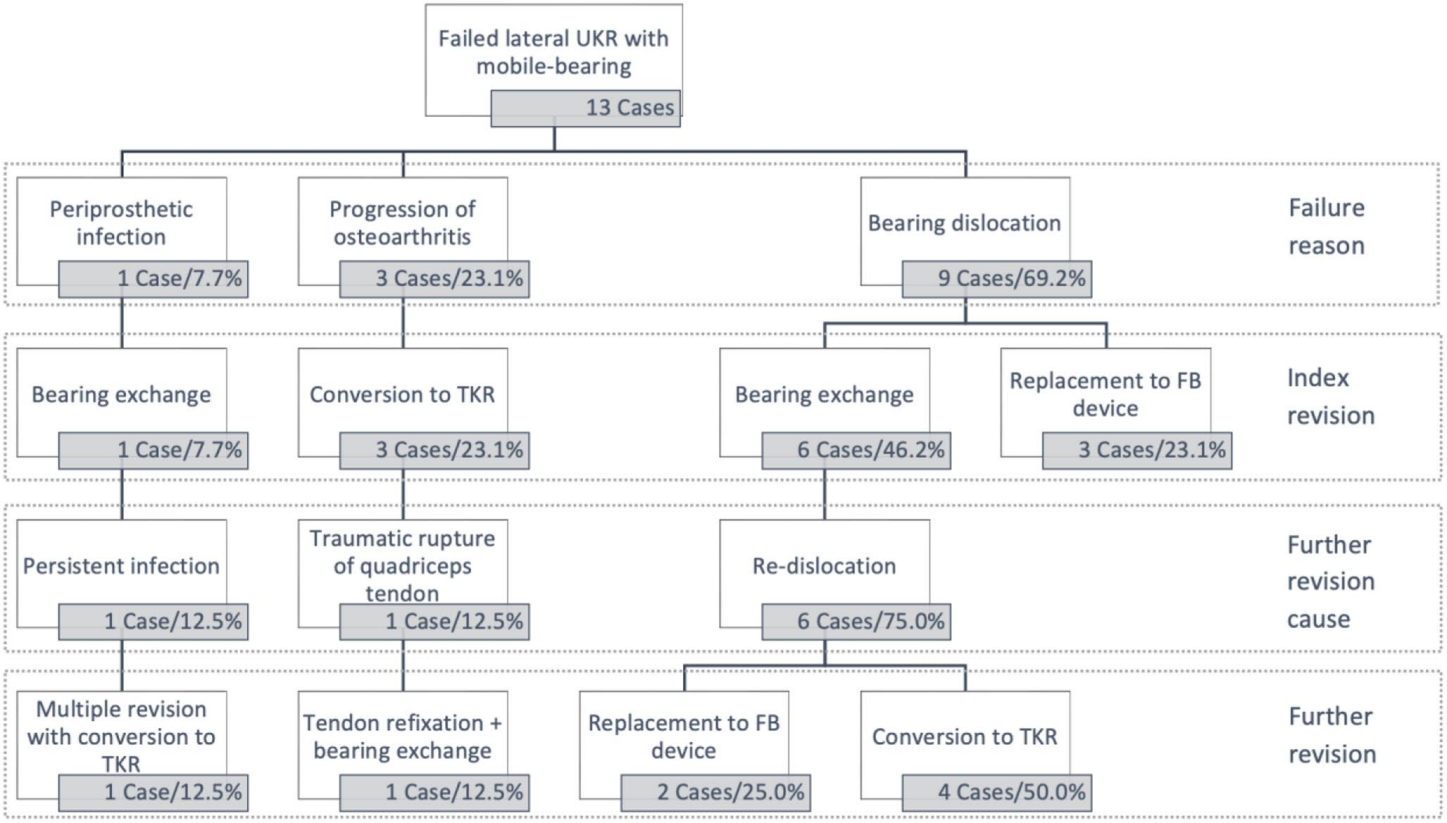
Flow chart of the study group demonstrating all failure causes and treatment strategies. UKR: unicompartmental knee replacement; TKR: Total knee replacement; FB: Fixed-bearing

All causes for revision and treatment strategies are illustrated in Fig. 1.

In eight patients (61.5%) at least one further revision surgery was necessary of which seven (87.5%) were initially revised by an isolated exchange of the bearing and one patient (12.5%) by conversion to TKR. The most common cause of re-revision was re-dislocation of the bearing (6 cases/75%). Additionally, one revision (12.5%) was due to persistent infection of the implant. Furthermore, one patient receiving TKR had a traumatic rupture of the quadriceps tendon leading to a further revision surgery in which the knee was explored and has shown instability in flexion. To gain more stability the bearing was exchanged to a thicker one.

All bearing dislocations were atraumatic and occurred in everyday movements.

**Fig. 2:**
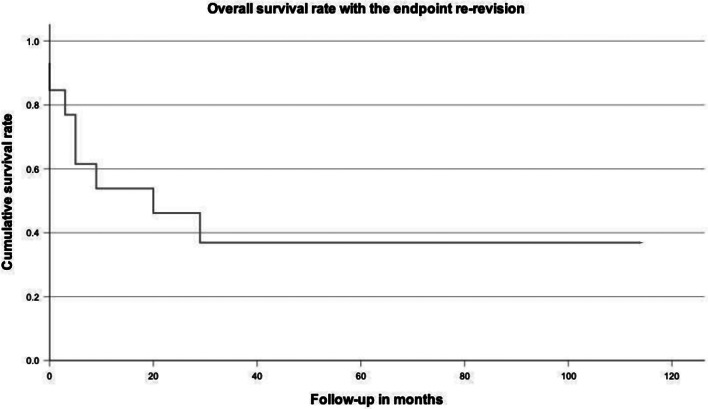
Kaplan-Meier survivorship curve of all thirteen patients for “re-revision” as the endpoint. The overall cumulative survival rate with the endpoint “re-revision” was 38.5% at a mean follow-up of 30.3 months

The cumulative survival rate with the endpoint “re-revision” was 38.5% at 30.3 months (Fig. 2). Survival rates subdivided for different treatment strategies with the endpoint “re-revision” showed heterogenous results (Table 2).

**Fig. 3:**
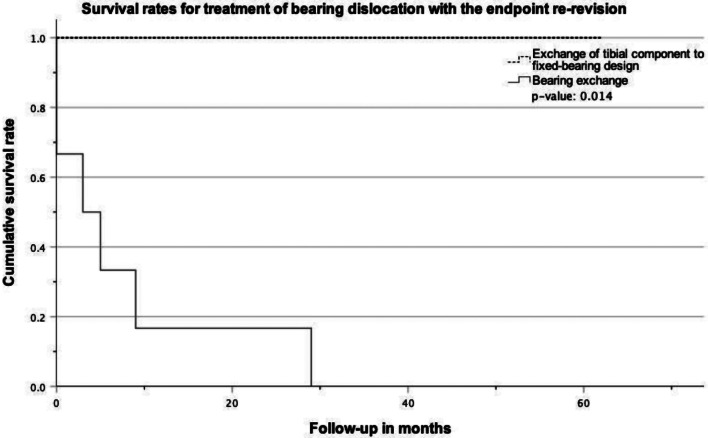
Kaplan-Meier survivorship curve for “re-revision” as the endpoint in patients treated for bearing dislocation with either exchange of the bearing or exchange of the tibial component to a fixed-bearing design. The log rank test showed a significant difference between both groups (p-value: 0.014)

In all nine patients with bearing dislocation as reason for index revision, treatment was either conducted with isolated bearing exchange (6 cases/0% survival rate with a mean FU of 8.4 ± 11 months) or exchange of the tibial component to a FB design (3 cases/ 100% survival rate with a mean FU of 47 ± 17.3 months). Survival rates between these two groups with the endpoint “re-revision” differed significantly in the log rank test (*p* = 0.014, Fig. 3).

**Fig. 4:**
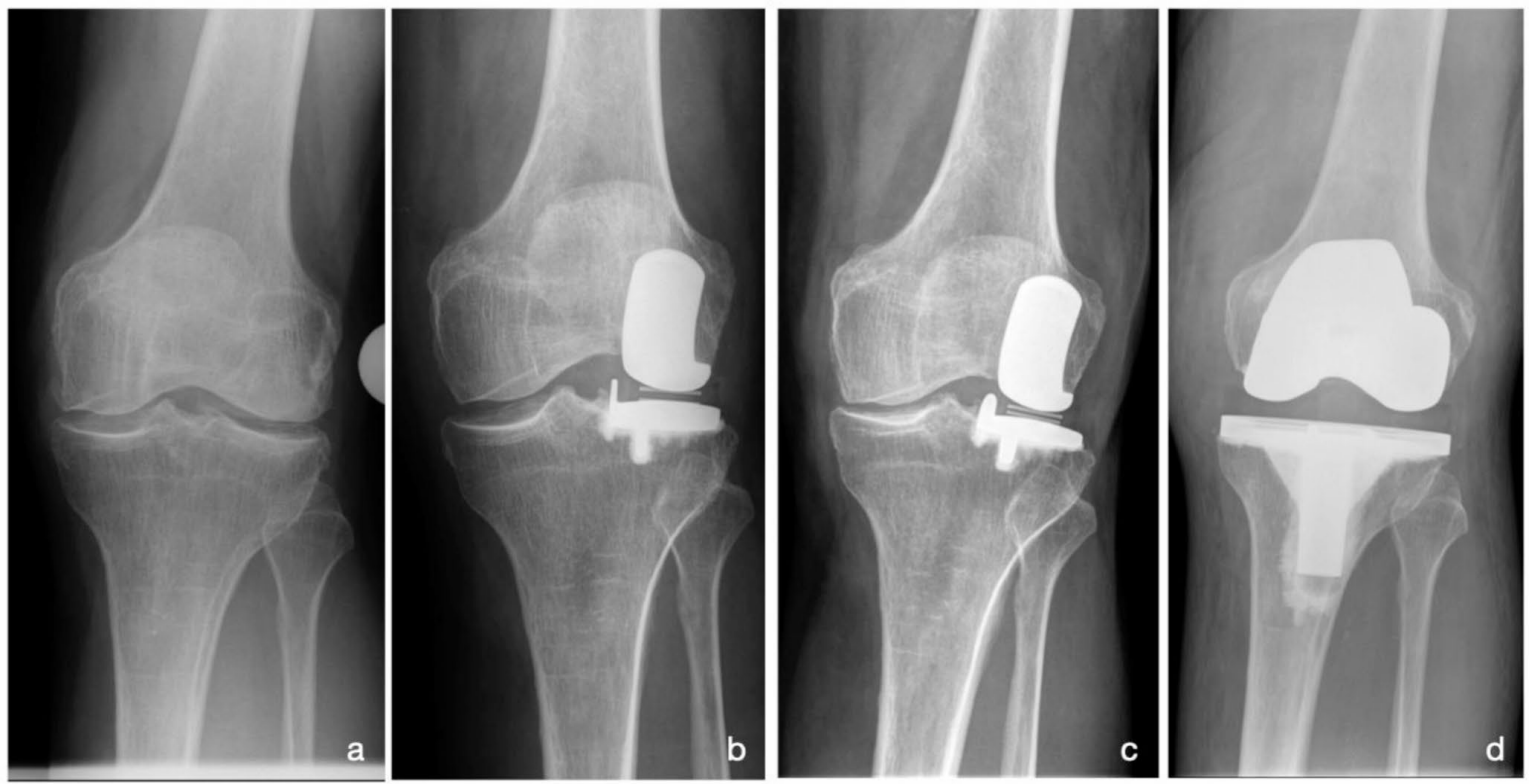
Case history of a 75-year-old male patient who developed progressive medial OA following the implantation of a lateral UKR (**c**), which ultimately required conversion to TKR 27 months after the initial surgery (**d**). As shown in radiograph b, the tibial bone resection appears to be very small, possibly causing an overcorrection of the knee alignment that may have contributed to the early progression of medial OA. Although the presented radiographs suggest preexisting OA in the medial compartment, the intraoperative assessment during the index surgery confirmed the indication for lateral UKR. **a**: Weight-bearing AP x-ray of the left knee prior to the initial surgery. **b**: Postoperative AP x-ray shortly after UKR. **c**: AP x-ray 27 months after lateral UKR, showing significant medial OA progression. **d**: AP x-ray after revision surgery to TKR. AP: antero-posterior, OA: osteoarthritis, TKR: total knee replacement, UKR: unicompartmental knee replacement

**Fig. 5:**
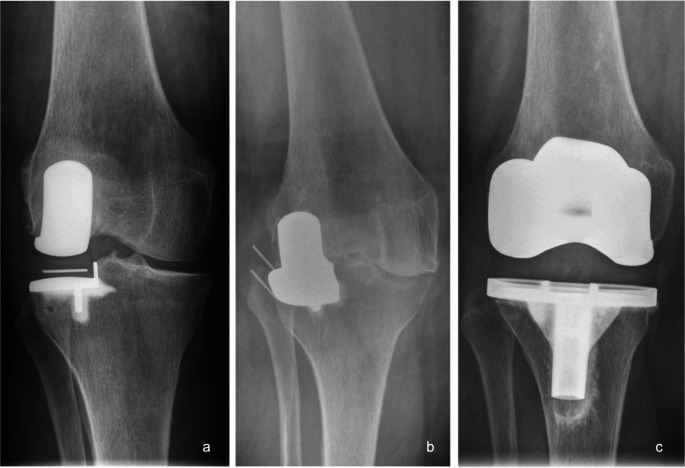
Case history of a 74-year-old female patient who experienced an atraumatic bearing dislocation while standing up from a chair, 16 months after receiving a lateral  UKR. Revision surgery was performed, during which the bearing was exchanged from size 7 to size 8. Thirteen months later, a second bearing dislocation occurred (**b**), necessitating conversion to a TKR (**c**), **a**: AP x-ray shortly after lateral UKR, **b**: x-ray after the second dislocation, showing lateral displacement with associated movement limitation of the knee, **c**: AP x-ray after revision surgery to TKR, AP: antero-posterior, TKR: total knee replacement, UKR: unicompartmental knee replacement

**Fig. 6:**
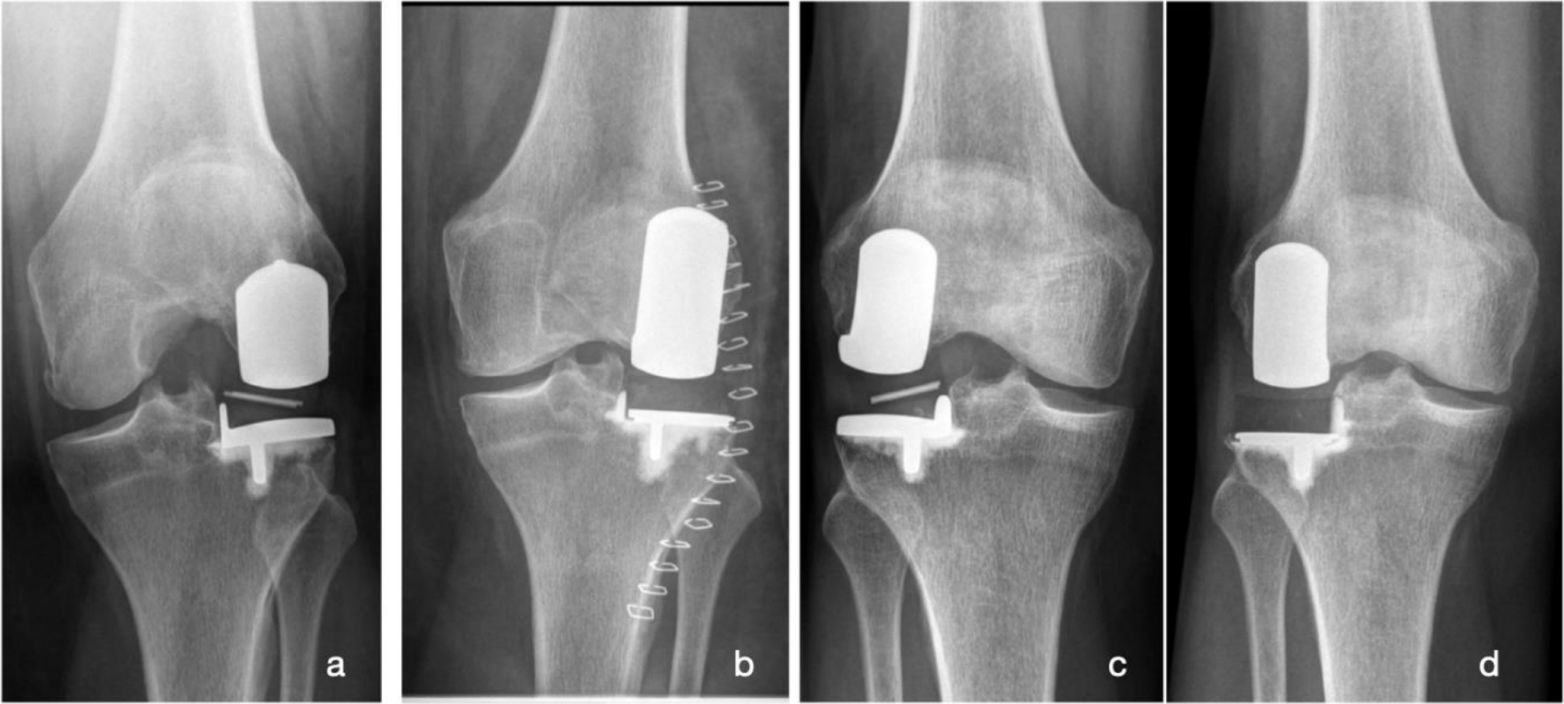
Radiographs of two female patients who have experienced medial bearing dislocation after 46 (**a**) and 60 (**c**) months, respectively. Both patients received an exchange of the tibial component to a fixed-bearing design (**b**: Oxford Fixed Lateral; **d**: Vanguard M Partial). **a** and c: AP x-rays with a dislocated bearing into the notch. **b** and **d**: Postoperative AP x-rays after revision to a fixed-bearing design. AP: antero-posterior

At last FU, eight patients (61.5%) were converted to TKR whereas five patients (38.5%) retained lateral UKR by isolated conversion to a FB tibial component. No patient needed a TKR revision-system to compensate for bone defect or ligament instability (Figs. 4, 5, 6).

**Table 2:** Survival rates of different treatment choices for revision surgery with the endpoint “re-revision”

Treatment choice for revision	Number of events	Survival rate with the endpoint re-revision (%)	Mean follow-up in months ± SD
Exchange to unconstrained TKR	1	66.7	62.3 ± 54.7
Exchange of tibial component to FB-design	0	100	47.0 ± 17.3
Bearing Exchange	7	0	9.4 ± 11.1

FB: fixed-bearing; TKR: Total knee replacement; SD: Standard deviation

Most common direction of bearing dislocation at the time of index revision surgery was medial (4 cases/44.4%) followed by anterior (3 cases/33.3%) and lateral direction (2 cases/22.2%). The direction in all 6 patients with re-dislocation happened to be the same as the first dislocation (2x medial, 2x anterior, 2x lateral).

## Discussion

The primary finding of this study is that the isolated bearing exchange, following a bearing dislocation in lateral MB-UKR, does not effectively prevent subsequent dislocations. In contrast, this strategy was associated with a 100% failure rate, with re-revision as the endpoint. The cause of bearing dislocation in lateral MB-UKR is only partly understood and continues to be a subject of current research [10–12]. The results of this study indicate that the risk factors for atraumatic bearing dislocation persist after bearing exchange, potentially making re-dislocation inevitable. These findings contradict the results and recommendations from the developer center, where bearing exchange proved successful in preventing re-dislocation in most cases [8, 13, 14].

With the increasing utilization of UKR over the last decades, the necessity for revision surgery in cases of failed UKR has become more frequent [15]. Treatment strategies for failed medial UKR typically involve conversion to unconstrained TKR or revision TKR systems, and in select cases, bi-unicompartmental knee replacement due to OA progression [15–18]. In contrast, the efficacy of revision surgery for failed lateral MB-UKR was not reported beside of bearing replacement from the developer center [13]. Despite limited sample size in this study, both the conversion to TKR and the isolated replacement of the tibial component are feasible even in the absence of severe bone defects. Especially isolated replacement of the tibial component from a MB to a FB design in lateral UKR has shown promise as a suitable approach for addressing recurrent dislocation. Given that most bearing dislocations occur in the short term, it can be presumed that the criteria for UKR are still applicable, thus favoring the retention of UKR as the preferred treatment option [5]. While this case series may be insufficient to conclusively establish the safety of the employed treatment strategies, it does showcase potential strategies for managing failure in lateral MB-UKR.

The progression of OA in the medial or patellofemoral joint is already recognized as the main cause of revision in the long-term for lateral UKR [3]. In this case series, three patients underwent revision surgery due to short-term OA progression, potentially attributable to overcorrection of the valgus deformity [19, 20].

In this study population, revision to TKR was achieved without resorting to the utilization of constrained TKR systems. Citak et al. reported more cases of failed lateral UKR being converted to constrained than to unconstrained TKR [21]. Recognizing the feasibility of revising lateral UKR to unconstrained TKR holds significance in patient education and decision-making. Surgeons who discourage patients from opting for UKR, because of the higher revision rates compared to TKR should know that revision to unconstrained TKR is still possible after failure of UKR [22].

This study carries certain limitations. The most notable among these is the small sample size and the retrospective nature of the study design. The inclusion of multi-center studies could provide a larger pool of cases involving failed lateral UKR. Notwithstanding, the principal objective of this study was to offer a comprehensive overview of failure patterns and potential treatment outcomes for an exceedingly rare clinical scenario within a non-designer center, rendering it a unique contribution to date.

Furthermore, patient-reported outcome measurements were not collected, as they did not align with the study’s primary objectives. Additionally, the occurrence of a few patient fatalities during the study period would further diminish the available pool of patients in the study group for clinical assessment, rendering any comparison scientifically untenable. Nonetheless, a clinical evaluation of the different revision strategies for lateral UKR remains essential to facilitate informed decision-making in the future.

## Conclusion

Addressing the issue of recurrent bearing dislocation in lateral MB-UKR demands a more comprehensive intervention than a simple bearing exchange. Optimal strategies include substituting the tibial component with an FB design or the conversion to TKR. Importantly, successful revision surgery for lateral UKR can be achieved without necessarilyaddressing concurrent bone defects or instability concerns.

## Data Availability

The datasets used and analyzed during the current study are available from the corresponding author on reasonable request.

## Abbreviations

ACL: Anterior cruciate ligament
BMI: Body mass index
FB: Fixed-bearing
FU: Follow-up
LCL: Lateral collateral ligament
MB: Mobile-bearing
MCL: Medial collateral ligament
MIS: Minimally invasive surgical
OA: Osteoarthritis
SD: Standard deviation
TKR: Total knee replacement
UKR: Unicompartmental knee replacement
